# Urinary tract infection is associated with hypokalemia: a case control study

**DOI:** 10.1186/s12894-020-00678-3

**Published:** 2020-07-20

**Authors:** Ai-Ling Shen, Hsiu-Li Lin, Hsiu-Chen Lin, Yuan-Fu Tseng, Chien-Yeh Hsu, Che-Yi Chou

**Affiliations:** 1 Department of Neurology, Sijhih Cathay General Hospital, No.2, Ln. 59, Jiancheng Rd., Sijhih Dist, New Taipei City, 221 Taiwan; 2 Graduate Institute of Biomedical Informatics, Taipei Medical University, No. 252, Wu-Xing Street, Taipei, 110 Taiwan; 3 Department of Pediatrics, School of Medicine, College of Medicine, Taipei Medical University, No. 252, Wu-Xing Street, Taipei, 110 Taiwan; 4 Department of Laboratory Medicine, Taipei Medical University Hospital, No. 252, Wu-Xing Street, Taipei, 110 Taiwan; 5 Department of Information Management, National Taipei University of Nursing and Health Science, No. 365, Mingde Rd, Taipei, 112 Taiwan; 6 Division of Nephrology, Asia University Hospital, NO 222, Fuxin Rd, Wufeng Dist, Taichung, 413 Taiwan; 7 Department of Post-baccalaureate Veterinary Medicine, Asia University, NO 222, Fuxin Rd, Wufeng Dist, Taichung, 413 Taiwan; 8 Division of Nephrology, China Medical University Hospital, Tacihung, Taiwan

**Keywords:** UTI, Hypokalemia, Recurrent UTI, Comorbidities, Diarrhea, Thiazides, Sulfonamides

## Abstract

**Background:**

Hypokalemia is a common clinical problem. The association between urinary tract infection (UTI) and hypokalemia is not clear. Hypokalemia is common in patients with UTI in clinical observation. The aim of the study is **t**o determine if UTI is associated with hypokalemia.

**Methods:**

Patients hospitalized with UTI and the control group were retrieved from the Longitudinal Health Insurance Database 2005. The control group was patients hospitalized with other reasons and were matched for the confoundings of UTI and hypokalemia. We analyze the risk of hypokalemia using logistic regression and calculate the odds ratio (OR) and 95% confidence interval (CI) of OR.

**Results:**

We analyzed 43,719 UTI patients and control patients. Hypokalemia was found in 4540 (10.4%) patients with UTI and 1842 (4.2%) control patients. The percentage of patients with hypokalemia was higher in UTI patients (chi-square, *p* < 0.001). UTI was associated with hypokalemia and the odds ratio (OR) was 2.27 [95% confidence interval (CI): 2.17–2.41]. Cerebrovascular accident, chronic obstructive pulmonary disease, hypertension, congestive heart failure, diarrhea, medications including thiazides, sulfonamides, xanthines, and laxatives were independently associated with hypokalemia. Recurrent UTI was associated with hypokalemia in UTI patients (OR: 1.13, 95% CI: 1.05–1.23, *p* < 0.001).

**Conclusions:**

Urinary tract infection is associated with hypokalemia among inpatients. The association is independent of patients’ comorbidities and medications. Recurrent UTI is associated with increased hypokalemia in UTI patients.

## Background

Hypokalemia is a common electrolyte abnormality in hospitalized patients. Unrecognized hypokalemia may trigger arrhythmias [1] and is associated with increased mortality in patients with chronic diseases such as heart failure, diabetes, or chronic kidney disease [2, 3]. Urinary tract infection (UTI) is a common bacterial infection in women and the elderly [4–6]. Clinical manifestations of UTI include dysuria, frequency, urgency, fever, nausea, vomit, and gross hematuria. It may not be surprised that hypokalemia may be occurred because of increased potassium loss after vomit and fever. However, the association between UTI and hypokalemia is not clear [7–11]. We observed that hypokalemia is common among UTI patients in clinical settings. This study was conducted to determine the association between UTI and hypokalemia. Inpatients were selected because serum potassium is rarely measured in outpatient settings as UTI has not been considered as a risk factor for hypokalemia.

## Methods

We extracted data from the Longitudinal Health Insurance Database 2005 (LHID2005) as prescribed in the previous study [12]. The dataset used in this study is a de-identified secondary data and is released to the public for research purposes. A review of the institutional review board is not required. All inpatients with UTI between 1 January 2000 and 31 December 2008 were extracted. The first hospitalization for UTI was selected in the patients with more than one episode of UTI. The control patients were patients who are hospitalized for other reasons with matching for confoundings at a 1:1 ratio. The confoundings include cerebrovascular accident (CVA), chronic obstructive pulmonary disease (COPD), diabetes mellitus (DM), hypertension (HTN), acute kidney injury (AKI), chronic kidney disease (CKD), Cushing syndrome, hyperthyroidism, leukorrhea, medications, and urethral catheterization. These confoundings were used because they are associated with UTI or hypokalemia in the literature.

The patients hospitalized with UTI were identified with ICD-9-CM code 599 on discharge. Patients on dialysis were excluded in this study because pyuria is common in auric dialysis patients. The development of hypokalemia was identified using ICD-9-CM code (276.8) in the same hospitalization. The comorbidities diagnosed before the episode of UTI in at least 3 visits were retrieved. The comorbidities include CVA (ICD-9-CM code 430–436 and 438), COPD (490–496), DM (250), HTN (401–405), CHF (428), AKI (584.9), CKD (585), Cushing’s syndrome (255.0), hyperthyroidism (242), hypothyroidism (244), alkalosis (276.3), diarrhea (787.91), malnutrition (269.9, 262, 263), poor intake (783.0), magnesium disorder (275.2), and leukorrhea (616 and 623.5). Urethral catheterization in 1 week before hospitalization was identify using procedure code 47013–47014. Recurrent UTI was defined as individuals who had more than two episodes of UTI in 6 months or three episodes in 1 year [13]. Medications used for more than 14 days in 1 month before the UTI diagnosed were extracted. Medications include potassium-sparing diuretics (ATC code C03D, C03E), thiazides (C03A, C03B), sulfonamides (C03CA01, C03CA02), beta-2-adrenoreceptor agonists (R03CC), xanthines (R03DA, R03DB), antibiotics (J01CA, J01CR, J01GB, A01AB04), meropenem (J01DH02), insulin (A10), steroids (H02AB), potassium binders (V03AE01), and laxatives (A06AG07).

### Statistical analysis

Data are expressed as number (percentage), or mean (standard deviation) where appropriate. Chi-square tests and *t*-test was used to test the differences of variables between two groups. The association of hypokalemia and UTI was estimated using univariable logistic regression. Variables with a *p* < 0.05 in univariable logistic regression were further analyzed by multivariable logistic regression. The odds ratios (ORs) and 95% confidence interval (CIs) of ORs were calculated. All statistical analyses were carried out using SAS statistical package, version 9.3 (SAS, Inc., NC, USA). All comparison tests were two-sided and a *P*-value of less than 0.05 was considered as statistically significant.

## Results

A total of 43,719 inpatients with UTI and matched control patients were analyzed (Table 1). 4540 (10.4%) UTI patients and 1842 (4.2%) control patients had hypokalemia (*p* < 0.001, chi-square test). The duration of hospitalization was longer in UTI patients 12.7 ± 24.8 days vs 7.4 ± 14.5 days in the control patients (*p* < 0.001). The average age of UTI patients was 63.5 ± 17.7 years old and 63.6 ± 18.2 years old in the control patients (*p* = 0.492). 39.3% of UTI patients and 39.2% of control patients were male. The percentage of patients with different comorbidities were similar in UTI patients and control patients after matching. The percentage of CVA was 28.8% in UTI patients and 28.4% in control patients. COPD 30.9 and 30.7%, DM 33.8 and 33.4%, HTN 59.2 and 58.9%, CHF 14.1 and 14.0%, AKI 0.3 and 0.3%, CKD 7.2 and 7.2%. Diarrhea, poor intake, and malnutrition were identified in 0.1% of the patients. The percentage of patients on potassium-sparing diuretics was 0.1% in UTI patients and 0.2% in control patients. Thiazides 0.6 and 0.6%, sulfonamides 0.4 and 0.5%, beta-2-adrenoreceptor agonists 0.7 and 0.7%, xanthiums 4.1 and 4.1%, antibiotics 0.7 and 0.7%, insulin 1.7 and 1.7%, steroids 1.5 and 1.5%, potassium binders 0.2 and 0.2%, laxatives 10.2 and 10.1%. The percentage of patients with urethral catheterization was 6.7% in both groups.

**Table 1:** Clinical characteristics of patients with and without urinary tract infection (UTI)

Factor	UTI *N* = 43,719	Control *N* = 43,719	*P*
Hypokalemia n (%)	4540 (10.4)	1842 (4.2)	< 0.001
Duration of hospitalization (day)	12.7 ± 24.8	7.4 ± 14.5	< 0.001
Age	63.6 ± 18.2	63.5 ± 17.7	0.492
Male n (%)	17,177 (39.3)	17,152 (39.2)	0.87
Comorbidity n (%)			
CVA	12,574 (28.8)	12,412 (28.4)	0.23
COPD	13,519 (30.9)	13,428 (30.7)	0.51
DM	14,776 (33.8)	14,615 (33.4)	0.25
HTN	25,860 (59.2)	25,752 (58.9)	0.46
CHF	6154 (14.1)	6128 (14.0)	0.81
AKI	119 (0.3)	117 (0.3)	0.95
CKD	3162 (7.2)	3125 (7.1)	0.64
Cushing syndrome	283 (0.6)	279 (0.6)	0.90
Hyperthyroidism	660 (1.5)	654 (1.5)	0.89
Leukorrhea	350 (0.8)	332 (0.8)	0.51
Diarrhea	59 (0.1)	62 (0.1)	0.85
Poor intake	40 (0.1)	46 (0.1)	0.59
Malnutrition	22 (0.05)	25 (0.06)	0.77
Medications n (%)			
Potassium-sparing diuretics	63 (0.1)	75 (0.2)	0.35
Thiazides	261 (0.6)	283 (0.6)	0.37
Sulfonamides	175 (0.4)	208 (0.5)	0.10
Beta-2-adrenoreceptor agonists	176 (0.4)	204 (0.5)	0.17
Xanthiums	1777 (4.1)	1782 (4.1)	0.94
Antibiotics	298 (0.7)	296 (0.7)	0.97
Insulin	752 (1.7)	763 (1.7)	0.79
Steroids	648 (1.5)	663 (1.5)	0.69
Potassium binders	105 (0.2)	105 (0.2)	–
Laxatives	4458 (10.2)	4412 (10.1)	0.61
Urethral catheterization	3034 (6.7)	3034 (6.7)	–

*CVA* cerebrovascular accident, *COPD* chronic obstructive pulmonary disease, *DM* diabetes mellitus, *HTN* hypertension, *AKI* acute kidney injury, *CKD* chronic kidney disease, *CHF* congestive heart failure

UTI was associated with hypokalemia in univariable and multivariable logistic regression (Table 2). The ORs were 2.48 (95% CI: 2.33–2.64, *p* < 0.001) in univariable logistic regression and 2.27 (95% CI: 2.13–2.41, *p* < 0.001) in multivariable logistic regression. Factors associated with hypokalemia in univariable logistic regression include age, CVA, COPD, DM, HTN, CHF, CKD, diarrhea, potassium-sparing diuretics, thiazides, sulfonamides, beta-2-adrenoreceptor agonists, xanthines, steroids, and laxatives. DM, CKD, beta-2-adrenoreceptor agonists, and steroids were not significantly associated with hypokalemia in multivariable logistic regression. The ORs of age was 1.01 (95% CI: 1.01–1.01) for every one additional year, CVA 1.29 (95% CI: 1.20–1.39), COPD 1.47 (95% CI: 1.37–1.57), HTN 1.56 (95% CI: 1.44–1.68), CHF 1.40 (95% CI: 1.28–1.53), diarrhea 2.25 (95% CI: 1.24–3.82), thiazides 1.57 (95% CI: 1.21–2.00), sulfonamides 1.44 (95% CI: 1.03–1.97), xanthines 1.44 (95% CI: 1.22–1.69), and laxatives 1.32 (95% CI: 1.16–1.52).

**Table 2:** Factors associated with hypokalemia in univariable and multivariable logistic regression

Factors	Univariable			Multivariable		
	ORs	95%	CIs	ORs	95%	CIs
UTI	2.48	2.33	2.64	2.27	2.13	2.41
Age	1.03	1.03	1.03	1.01	1.01	1.01
Comorbidity						
CVA	2.29	2.14	2.44	1.29	1.20	1.39
COPD	2.21	2.08	2.34	1.47	1.37	1.57
DM	1.56	1.46	1.66	1.02	0.95	1.09
HTN	2.62	2.46	2.79	1.56	1.44	1.68
CHF	2.49	2.28	2.71	1.40	1.28	1.53
CKD	1.42	1.21	1.66	0.98	0.84	1.15
Diarrhea	2.29	1.28	3.82	2.25	1.24	3.82
Medications						
Potassium-sparing diuretics	1.96	1.12	3.19	1.24	0.69	2.08
Thiazides	1.98	1.53	2.51	1.57	1.21	2.00
Sulfonamides	2.26	1.64	3.06	1.44	1.03	1.97
Beta-2-adrenoreceptor agonists	2.04	1.48	2.76	0.91	0.64	1.26
Steroids	1.57	1.29	1.89	1.12	0.91	1.37
Xanthines	2.38	2.05	2.74	1.44	1.22	1.69
Laxatives	2.07	1.81	2.35	1.32	1.16	1.52

*UTI* urinary tract infection, *CVA* cerebral vascular accident, *COPD* chronic obstructive pulmonary disease, *DM* diabetes mellitus, *CHF* congestive heart failure, *CKD* chronic kidney disease

Recurrent UTI was significantly associated with hypokalemia in UTI patients (Table 3). The ORs were 1.31 (95% CI: 1.21–1.41) in univariable logistic regression and 1.13 (95% CI: 1.05–1.23) in multivariable logistic regression. Patient’s age, CVA, COPD, HTN, CHF, thiazides, sulfonamides, xanthines, and laxatives were independently associated with hypokalemia in UTI patients in univariable logistic regression, but diarrhea was not. The OR of age in the multivariable logistic regression was 1.01 (95% CI: 1.01–1.01), CVA 1.16 (95% CI: 1.08–1.24), COPD 1.45 (95% CI: 1.35–1.55), HTN 1.42 (95% CI: 1.31–1.53), CHF 1.41 (95% CI: 1.31–1.53), xanthines 1.42 (95% CI: 1.25–1.62), and laxatives 1.29 (95% CI: 1.17–1.41).

**Table 3:** Risk of hypokalemia among patients with urinary tract infection

Factors	Univariable			Multivariable		
	ORs	95%	CIs	ORs	95%	CIs
Recurrent UTI	1.31	1.21	1.41	1.13	1.05	1.23
Age	1.02	1.02	1.03	1.01	1.01	1.01
Comorbidity						
CVA	1.71	1.60	1.82	1.16	1.08	1.24
COPD	2.08	1.95	2.21	1.45	1.35	1.55
HTN	2.20	2.05	2.36	1.42	1.31	1.53
CHF	2.16	2.01	2.33	1.41	1.31	1.53
Diarrhea	1.61	0.85	2.82	1.82	0.95	3.23
Medications						
Thiazide	1.60	1.34	1.90	1.16	0.97	1.38
Sulfonamides	1.74	1.52	1.98	1.14	0.99	1.31
Xanthines	2.29	2.02	2.59	1.42	1.25	1.62
Laxatives	1.80	1.65	1.97	1.29	1.17	1.41

*UTI* urinary tract infection, *CVA* cerebral vascular accident, *COPD* chronic obstructive pulmonary disease, *DM* diabetes mellitus, *CHF* congestive heart failure

## Discussion

In this case-control cross-sectional study, we demonstrated the association between UTI and hypokalemia among inpatients. This finding is supported by the increased OR of hypokalemia in the multivariable logistic regression. The association between hypokalemia and UTI is independent of comorbidities and mediations. Besides, recurrent UTI is also associated with hypokalemia in UTI patients. Hypokalemia in UTI may explain the cardiac arrest following infectious disease in the previous study [14]. Inpatients with UTI were selected in this study because the serum potassium was more likely to be measured in inpatient settings. The development of hypokalemia in the patients with UTI may be explained by multiple factors including renal [9, 15, 16], medications [17], gastrointestinal loss. Medications including thiazides, sulfonamides, xanthines, and laxatives were independently associated with hypokalemia. We hypothesized that hypokalemia developed because of renal potassium loss. The renal tubule potassium resorption was impaired after tubule injury in UTI and therefore the urine potassium loss is increased [18, 19]. We measured the urine potassium of several UTI patients (data not shown) and the urine potassium secretion was increased in patients with UTI. More studies are needed to verify our findings.

Because hypokalemia may be related to multiple comorbidities, we include comorbidities that may be associated with both UTI [20, 21] and hypokalemia [22] in the analysis. DM, CKD, potassium-sparing diuretics, beta-2-adrenoreceptor agonists, and steroids were associated with hypokalemia in univariable regression but not in multivariable regression. This suggests that these may be not major contributors to hypokalemia in UTI patients. A few patients (0.1%) had diarrhea, malnutrition, or poor intake in our study. We suspected these conditions were underdiagnosed because physicians rarely coded these conditions in the diagnosis. Although the numbers of patients are limited, diarrhea was associated with hypokalemia in univariable and multivariable regressions. Duration of hospitalization, alkalosis, malnutrition, poor intake, magnesium disorder, and meropenem [23] were analyzed and was not linked to hypokalemia.

### Limitations

The limitations to the study include: First, LHID only recorded the ICD-9-CM code on discharge, it is not possible to discriminate community acquired hypokalemia and hypokalemia developed in hospital. There may be many factors which may cause hypokalemia in hospital. The causal relationship can not be determined. Some of the recurrent UTI may be caused by the hypokalemia that results in the elaboration of alkaline urine, bladder dysfunction, and urinary stasis [24]. Second, the serum potassium readings and symptoms associated with hypokalemia were not available in the LHID, we were not able to report the severity of hypokalemia in this study, and symptoms associated with hypokalemia. In our clinical practice, most of the hypokalemia was asymptomatic and potassium replacement was rarely acquired. Third, we hypothesized that the renal parenchyma injury in UTI may be responsible for the development of hypokalemia. Acute pyelonephritis may be associated with more severe renal parenchyma injury than lower UTI and is more likely to develop hypokalemia. However, we did not find a significant association between acute pyelonephritis and hypokalemia. We suspected that most acute pyelonephritis may be recorded as UTI in the LHID.

## Conclusion

Urinary tract infection is associated with an increased risk of hypokalemia and measurements of serum potassium should be considered in clinical practice. UTI and hypokalemia are associated with multiple chronic diseases and medications. The association of hypokalemia and UTI is independent of comorbidities and medications.

## Data Availability

The datasets used and/or analyzed during the current study are available from the corresponding author on reasonable request.

## Abbreviations

LHID: Longitudinal Health Insurance Database
ICD-9-CM: International Classification of Disease, Ninth. Revision, Clinical Modification
UTI: Urinary tract infection
CVA: Cerebral vascular accident
COPD: Chronic obstructive pulmonary disease
DM: Diabetes mellitus
CHF: Congestive heart failure
CKD: Chronic kidney disease
